# Anti-cancer effect of gallic acid in presence of low level laser irradiation: ROS production and induction of apoptosis and ferroptosis

**DOI:** 10.1186/s12935-020-1100-y

**Published:** 2020-01-13

**Authors:** Khatereh Khorsandi, Zahra Kianmehr, Zohreh hosseinmardi, Reza Hosseinzadeh

**Affiliations:** 1 Department of Photodynamic, Medical Laser Research Center, Yara Institute, ACECR, Tehran, Iran; 2 Department of Cellular and Molecular Biology (Biochemistry), Faculty of Biological Science, North Tehran Branch, Islamic Azad University, Tehran, Iran; 3 Department of Medical Laser, Medical Laser Research Center, Yara Institute, ACECR, Tehran, Iran

**Keywords:** Gallic acid, Low level laser irradiation, Breast cancer, Melanoma cancer, Apoptosis, Ferroptosis

## Abstract

**Background:**

There are different treatments for breast cancer and melanoma that mostly have some side effects. One of the therapeutic strategies is the use of natural components. Phenol components are a class of antioxidants in plants that have many biological functions like anticancer effects. Gallic acid (GA) is a natural polyhydroxy phenolic compound and commonly found in various foods. In the present study, GA effects alone and in combination with low-level laser irradiation on human dermal fibroblast cell line (HDF), human non-tumorigenic breast epithelial cell line (MCF10A), breast cancer cell line (MDA-MB-231) and melanoma cancer cell line (A375) was under the investigation.

**Methods:**

The normal and cancerous cell lines were exposed to 660 nm low-level laser with 3 J/cm^2^ for 90 s. Then, the cells were treated with different concentrations of GA for 24 h. In another study, the cell lines firstly were treated with GA and then exposed to low-level laser irradiation. The effects of GA and low-level laser on cell survival and apoptosis were examined using MTT assay, light microscopy, ROS production assay, fluorescence microscopy (AO/EB double staining) and flow cytometry.

**Results:**

The results showed that pre-treatment with low-level laser and then GA reduced the survival of breast cancer cells and melanoma more than the first treatment with GA and then low-level laser irradiation. Our findings showed that ROS production in cells treated with both low-level laser and GA was more than the cells treated with GA alone. The apoptosis and ferroptosis assays confirmed the MTT results which combination treatment with low-level laser and then GA increase the cell death probably via apoptosis and ferroptosis cell death mechanisms compared to GA alone.

**Conclusions:**

This study suggests that low-level laser irradiation alone is not able to cause death in human normal and cancerous cells. Preirradiation followed by GA treatment did not change the cell viability in human normal significantly but reduces the cell survival of cancer cells more than GA alone.

## Background

Breast cancer is the most common cancer in women that accounts for 33% of all cancers in women worldwide. Treatment of breast cancer often requires a multifactorial approach and may be performed with local therapy (such as surgery and radiation), systemic therapy (such as chemotherapy, hormonal therapy, and biologic or targeted treatments), or both [1, 2]. Breast cancer is a heterogeneous disease that is biologically diverse. Different types of the disease respond well to treatment. However, negative-triple breast cancer (TNBC) accounts for %15 of all breast cancers that do not respond well to treatment, and a high percentage of TNBC cancer deaths are due to metastasis [3–5]. Skin cancer is one of the most common cancers that are manageable and preventable, which is often overlooked. Skin cancer divided into melanoma and non-melanoma subgroups. Melanoma related to melanocyte cells. Melanoma is the most aggressive type of skin cancer and resistant to all kinds of treatments [6, 7]. Melanocyte differentiation-specific genes and their pigmentation are potential important indicators for melanoma. Melanoma is more common in women than in men, and it manifests itself in men in the trunk and in women in the feet. Clinically, the asymmetric and reddish-brown color of the melanoma noted irregular edges and associated with itching and bleeding [8–10].

**Fig. 1:**
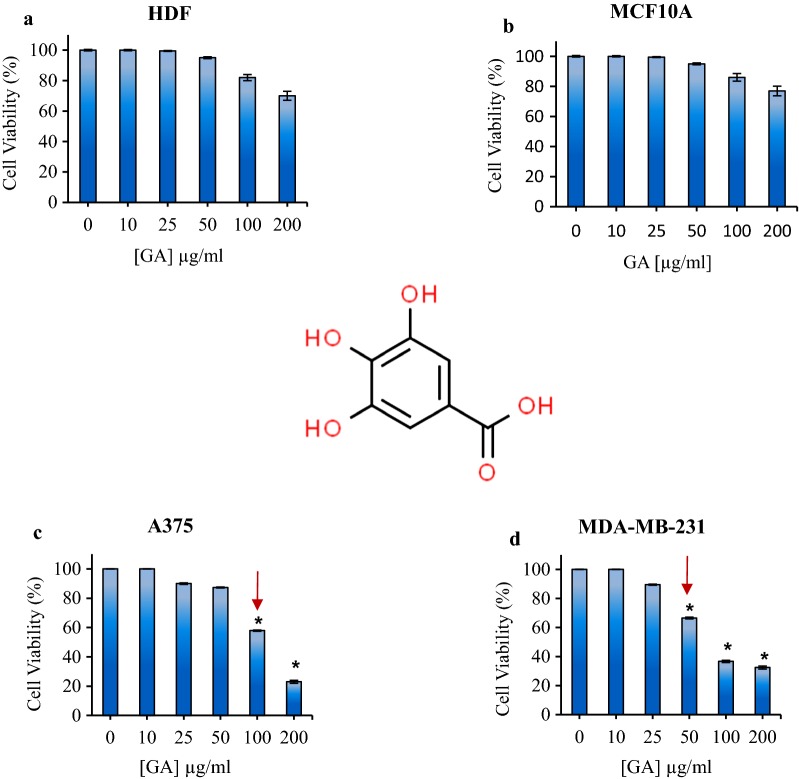
Schematic structure of Gallic acid (GA) chemical structure. The cell viability of **a** HDF fibroblast, **b** MCF10A normal breast **c** A375 melanoma and **d** MDA-MB-231 cells treated with different concentrations of gallic acid in dark condition. The arrows show the IC_50_. The results are expressed as mean ± SD (n = 3), *P < 0.05 compared with control (untreated) group

Phenolic compounds are secondary metabolites in plants that contain one or more aromatic rings containing hydroxyl groups. More than 8000 natural phenolic compounds have been identified to date. Phenolic compounds isolated from plants include simple phenols: flavonoids, ligands, tannins, xanthines, and coumarins [11, 12]. These phenolic compounds are known compounds that have anti-cancer activity, as a fighter against various diseases related to oxidative stress. Gallic acid (GA) is one of the known polyphenols in nature [13–15]. GA or 3,4,5- trihydroxy benzoic acid is an important compound against cancer with antioxidant properties [16, 17]. The chemical structure of GA was shown in Fig. 1a.

Among the advanced technologies, low-level laser irradiation is a relatively new method. The low-level laser irradiation from red to infrared (NIR) wavelength without significant thermal effects on cells, which treat a wide range of diseases including wound healing and tissue repair, reduce inflammation and relieve pain [18, 19]. Caro et al. showed that electron transfer chain systems and metal complexes in mitochondria, in particular the cytochrome c oxidase molecule, can act as the primary optical acceptor for the absorption of low-level laser light in the red and near-infrared spectrum [20]. Recent studies have shown that laser irradiation has increased the permeability of cells resulting in a more effective penetration of nutrients/drug into the cell [21, 22]. Therefore, photobiostimulation could has a great impact on treatment [23, 24].

Regulated cell death mechanisms other than apoptosis have emerged in recent years [25]. The term ferroptosis was introduced [26] to describe cell death induced by the compound erastin, which causes glutathione depletion through system X_c_^−^ inhibition and consequently glutathione peroxidase 4 (GPX4) inactivation. GPX4 functions to remove lipid peroxides generated in phospholipid membranes [27]. Ferroptosis may contribute to degenerative pathologies and might be therapeutically beneficial in some cancers [28].

Intracellular malondialdehyde (MDA) was quantified as an indicator of lipid peroxidation [29]. It showed an increase of MDA in cells is related to ferroptosis [30].

It has been said that ferroptosis is characterized biochemically by increased levels of lipid hydroperoxides and iron overload, iron-catalyzed generation of ROS, and lipid peroxidation [26, 31, 32], and by decreased level of GSH [33].

In this study, we consider the effects of GA on breast (MDA-MB-231) and melanoma (A375) cancer cell line in presence of low-level laser irradiation and evaluated the apoptosis and ferroptosis by determining the activity of GPX4 and MDA as key enzymes in ferroptosis cell death.

## Materials and methods

### Materials

Gallic acid, 3-(4, 5-dimethylthiazol-2-yl) 2, 5-diphenyltetrazoliumbromide (MTT), trypan blue solution 0.4%, acridine orange, ethidium bromide and dimethyl sulfoxide (DMSO) were obtained from Sigma-Aldrich (St Louis, MO, USA). Fetal bovine serum (FBS), phosphate buffer saline (PBS) and antibiotics were bought from Gibco (Gibco BRL). Dulbecco’s Modified Eagle Medium (DMEM) was received from Invitrogen (Invitrogen, Carlsbad, California, US). All the other reagents were purchased from Merck. Deionized (D.I.) water was used for the entire experiment.

### Cell culture

Breast cancer cell lines (MDA-MB-231), melanoma cancer cell line (A375) and human dermal fibroblast cell line (HDF) were obtained from the Institute of Pasture, Tehran, Iran. These cells were grown in DMEM medium supplemented with 10% FBS, 100 IU ml^−1^ penicillin, and 100 µg ml^−1^ of streptomycin and then incubated in a humidified incubator containing 5% CO_2_ at 37 °C. The human non-tumorigenic breast epithelial MCF10A cell line was purchased from the Institute of Pasture, Tehran and cultured in DMEM/F-12 supplemented with 10.0% FBS, 0.5 µg/ml of hydrocortisone, 10 µg/ml of insulin, 20 ng/ml of epidermal growth factor, 0.5 KU/ml of penicillin, 0.1 mg/ml of streptomycin, and 0.5 µg/ml of amphotericin B in 5.0% CO_2_ at 37 °C. For the experiments, the cells were removed by trypsinizing (trypsin 0.025%, EDTA 0.02%) and washed with PBS.

### Effect of different concentrations of GA on human normal and cancerous cells

Briefly, the normal and cancerous cells were seeded using fresh culture medium in 96 well plates and incubated under 5% CO_2_, at 37 °C for 24 h. Then, the cells were incubated using fresh cell culture medium containing different concentrations of GA (0, 10, 25, 50, 75, 100 and 200 μg/ml). After certain incubation time (24 h); the cells were washed by PBS solution. The colorimetric MTT assay was used to determine the viability of the cells. Each experiment was repeated three times.

### In vitro laser irradiation

Irradiation was performed with a red light source (660 nm; power density: 30 mW cm^−2^). The plates were divided into a control group that received no irradiation, and the treatment group that exposed to 660 nm low-level laser received an irradiation dose of 3 J/cm^2^ for 90 s. Irradiation was carefully timed and carried out in a dark laminar flux hood. The light source power-meter was done using power metric devices by the Electronics Research Institute at Sharif University of Technology (SUT), Optic laboratory, Tehran, Iran.

### Pre and post-treatment of normal and cancerous cells with low-level laser irradiation and GA

The normal and cancerous cells (1 × 10^4^ cells) were separately seeded in 96 well plates and incubated in 5% CO_2_ and 37 °C for 24 h. For the pre-treatment experiment, the cells were first irradiated as mentioned above section and then treated with fresh cell culture medium containing different concentrations of GA and incubated in 5% CO_2_ and 37 °C for 24 h. For the post treatment experiment, the cells were firstly treated with fresh cell culture medium containing different concentrations of GA and then one of the plates considers as control [no irradiation (dark)] and the other plate irradiated as mentioned above section and incubated in 5% CO_2_ and 37 °C for 24 h. Finally, in both experiments, the cells were washed with PBS and the cell viability was measured by MTT assay. Each experiment was repeated three times.

### MTT assay

Thiazolyl blue tetrazolium bromide (MTT) was used in the determination of cell survival as a colorimetric MTT assay. Cell viability can be measured as a function of the cell’s redox potential. Living cells convert the MTT compound to an insoluble formazan. The resulting formazan solubilized using dimethyl sulfoxide (DMSO) and its concentration determined using spectrophotometric methods. Briefly, the culture medium was removed and cells were incubated in medium containing 0.5 mg/ml of 3-(4, 5-dimethylthiazol-2-yl)-2,5-diphenyltetrazolium bromide for 4 h at 37 °C. The resulting purple formazan crystals dissolved in 100 μl DMSO and shacked for 15 min. The absorbance of samples was measured at 540 nm by an ELISA reader (Hyperion, Inc., FL, U.S.A.). Each experiment was repeated 3 times and data are represented as the mean ± SD.

### Inverted light microscopy and colony-forming assay

To investigate the morphology changes of MDA-MB-231 and melanoma A375 cancer cells after treatment with low-level laser irradiation and GA, the cells were exposed to irradiation at 660 nm for 90 s then were incubated with a dose of IC_50_ of GA for 24 h. Afterward, the cells were studied by a light inverting microscope at 40× magnification. For colony assay study the treated cells were collected by trypsinization and total numbers of cells were counted and 200 cells/plate were seeded. Following a 1-week incubation at 37 °C, colonies were stained with 0.5% crystal violet in methanol and the number of colonies was counted. The control was untreated cells kept for 24 h.

### Apoptosis induction by low-level laser irradiation and GA: AO/EB double staining and annexin V/PI flow cytometry analysis

For this experiment, the MDA-MB-231 and A375 cells were separately seeded in the petri dish, and after 24 h incubation in 5% CO_2_ and 37 °C, one petri considers as control (dark) and the other irradiated using red light irradiation for 90 s. After irradiation time, both petri were incubated using a fresh cell culture medium containing different concentrations of GA. After 24 h, the cells were pelleted, resuspended in 100 µl of PBS and were stained with Acridine Orange/Ethidium Bromide (AO/EB) according to published procedures [34]. The final concentrations of AO (Sigma, USA-A6014) and EB (Sigma, USA-E7637) were 0.1 and 0.25 mM, respectively. Morphological changes due to the induction of apoptosis were detected by fluorescence microscopy (BEL, Italy).

In order to determine the percentage of apoptotic cells in GA treated cells and compare it with the control cell, the cancer cells were stained with Annexin-V and propidium iodide (PI). For this purpose, cells were first exposed to low-level laser irradiation for 90 s at a wavelength of 660 nm and then were treated with GA at concentrations of 0 and IC_50_ GA (25 µg/ml in the case of MDA-MB-231 cells and 50 µg/ml in the case of A375 cells) for 24 h. The cells were then stained with propidium iodide (PI) and Annexin-V. The samples were incubated at 25 °C for 10 min in dark. Finally, the cells were analyzed using flow cytometry. Data analysis was performed using image j software and recorded into four zones Q1 to Q4. Q1 represents necrotic cells with specificity: PI^+^ Annexin-V^−^. Q2 represents late apoptotic cells with specificity: PI^+^ and Annexin-V^+^, Q3 represents live cells with specificity: PI^−^ and Annexin-V^−^ and Q4 represents early apoptotic cells with specificity: PI^−^ and Annexin-V^+^.

### ROS production in cancer cells after irradiation and treatment with GA

The intracellular accumulation of reactive oxygen molecules was measured by the 7.2-dichlorofluorescein diacetate (DCFH2-DA) assay. For this purpose, MDA-MB-231 breast and A375 melanoma cancer cells were cultured in approximately 10^6^ cells per petri dish. Cells were first exposed to low-level laser irradiation at 660 nm for 90 s and then the cells were treated with GA at concentrations of 0 and IC_50_ GA (25 µg/ml in the case of MDA-MB-231 cells and 50 µg/ml in the case of A375 cells) for 24 h. Cell culture was removed and the cells were incubated with 2 mM DCFH2-DA for 45 min in the dark. Then, the cells were washed with PBS and transferred to a flow cytometer for ROS testing. The data from the readings were analyzed with FlowJo 7.6.1 software and related charts are presented in the results section.

### Glutathione peroxidase

Glutathione peroxidase (Gl-Px) activity was evaluated by spectrophotometry using tert-butylperoxide as a substrate [35], monitoring the formation of oxidized glutathione, through the quantification of the oxidation of NADPH to NADP+ at 340 nm. The enzyme activity is expressed in mU/ml.

### Lipid peroxidation evaluation: determination of MDA

The MDA level was determined through thiobarbituricacid (TBA) method. The supernatant of cells was mixed with 1 ml of TBA (0.67%) and 3 ml of phosphoric acid (1%) and then placed in bathroom for 45 min [36]. After cooling, the products were extracted in 3 ml of normal butanol and centrifuged at 3000 rpm at (4 °C) for 10 min and the absorbance was measured by spectrophotometer at 532 nm.

### Statistical analysis

Statistical analysis was performed with student’s t-test (two tailed). All values are expressed as mean ± SD. *P* < 0.05 was considered as statistically significant.

## Results

### Effect of GA on normal and cancerous cells: dark cytotoxicity

In order to evaluate the cytotoxicity effect of GA in the absence of irradiation, the viability of treated cells after 24 h incubation with different concentrations of GA (0, 10, 25, 50, 75, 100 and 200 μg/ml) was determined at dark condition.

The cell viability of human dermal fibroblast cell line (HDF) in presence of GA did not significant change and in higher concentration at 100 µg/ml and 200 µg/ml, it slightly changed to 82% and 70%, respectively (Fig. 1a). In the case of human non-tumorigenic breast epithelial MCF10A cell, treatment with GA did not change and in higher concentration at 100 µg/ml and 200 µg/ml, it slightly changed to 86% and 77%, respectively (Fig. 1b).

The results of the effect of GA on melanoma A375 cell line in the absence of light showed that the survival of melanoma cancer cells decreased in the presence of GA and the cell viability was 23% at the concentration of 200 μg/ml (Fig. 1c). As is seen in the Fig. 1d in the absence of light and at 0, 10, 25, 50, 100 and 200 μg/ml of GA, the cell viability of MDA-MB-231 breast cancer cell line were 100%, 100%, 89.5%, 66.5%, 36.7% and 32.4%, respectively.

According to the obtained results, the half maximal inhibitory concentration (IC_50_) for GA on melanoma A375 cell line was about 100 µg/ml and for the MDA-MB-231 cell line, IC_50_ of GA was about 50 µg/ml after 24 incubation at dark condition.

### Pre and post low-level laser irradiation effect on GA cytotoxicity in normal and cancerous cells

**Fig. 2:**
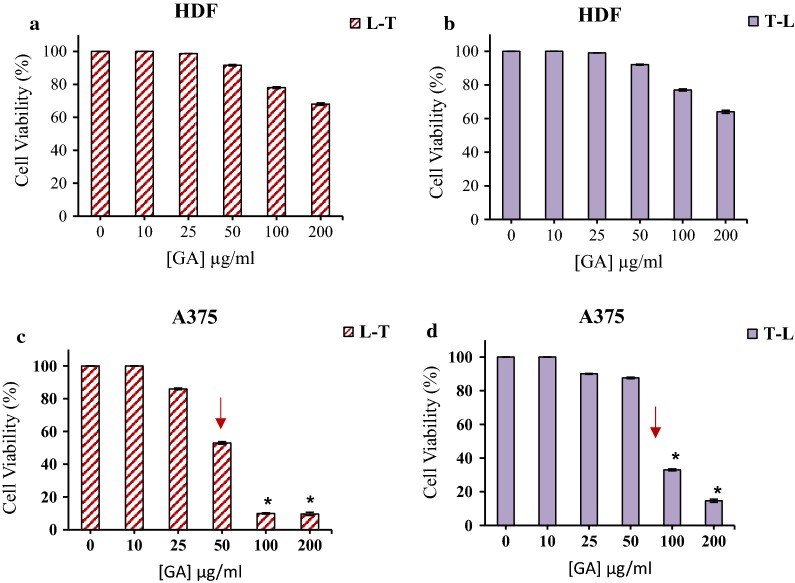
The cell viability of HDF treated with different concentrations of GA in pre (**a**), and post (**b**) red light irradiation. The cell viability of A375 cells treated with different concentrations of GA in pre (**c**), and post (**d**) red light irradiation. Low-level laser irradiation was used with 3 J/cm^2^ for 90 s. The results are expressed as the mean ± SD (n = 3), *P < 0.05 compared with control (untreated) group

The cytotoxicity effect of GA in the presence of pre and post low-level laser irradiation was evaluated in normal and cancerous cells. As can be seen in Fig. 2, in control samples contained PBS, there were no significant changes in comparison to the dark group which approves the light dose used at the irradiation time didn’t have any photo-toxicity in the absence of GA on human normal and cancerous cells.

The study of the effect of GA on HDF and A375 melanoma cancer cells by pre-irradiation at 660 nm with 3 J/cm^2^ energy showed that in the case of HDF cells the irradiation did not change the cell viability in compare to dark group (Fig. 2a, b). Low-level laser pretreatment decreased cell survival of A375 melanoma cells. The cell viability in pre- and post-irradiation respectively were 9.7% and 14.7% at 200 μg/ml of GA (Fig. 2c, d).

**Fig. 3:**
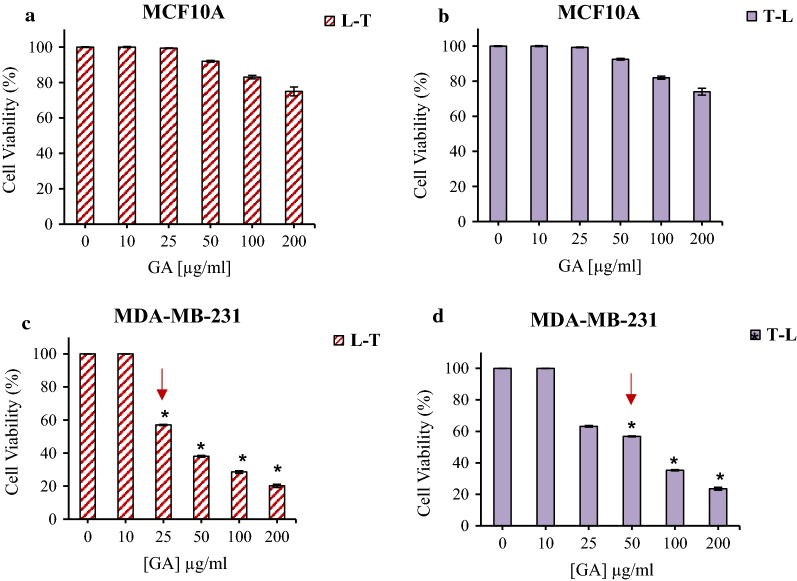
The cell viability MCF10A cells treated with different concentrations of GA in pre (**a**), and post (**b**) red light irradiation. The cell viability of MDA-MB-231 cells treated with different concentrations of GA in pre (**c**), and post (**d**) red light irradiation. Low-level laser irradiation was used with 3 J/cm^2^ for 90 s. The results are expressed as the mean ± SD (n = 3), *P < 0.05 compared with control (untreated) group

The MCF10A cell, pre and post treatment at 660 irradiation with 3 J/cm^2^ energy and GA did not show significantly cell viability difference compared to dark (control) group (Fig. 3a, b). In the case of breast cancer cells, in pretreatment at 660 irradiation with 3 J/cm^2^ energy, the cell viability decreases up to 20.13% at 200 μg/ml GA (Fig. 3c). The results of MDA-MB-231 cell line treatment with GA and then low- level laser irradiation showed that survival of breast cancer cells exposed to GA at 200 µg/ml was 12.4% (Fig. 3d).

As it can be seen the cell viability of normal cells (HDF and MCF10A) did not significant changed in compare to dark group (Fig. 1). From the results, it is clear that IC_50_ of GA on human melanoma (50 µg/ml) and breast cancer cell (25 µg/ml) lines was decreased by using pre-irradiation (Fig. 2). It could be suggested that in the presence of red light irradiation, phototoxic reactions sensitized cancer cells to GA and therefore, reduced the cell viability. It may be because of the role of red light irradiation in sensitizing cancer cells to treatment. As GA can act as an antioxidant, using it before the irradiation could neutralize the effect of red light irradiation in sensitizing the cancer cells to therapy.

### Different pre-irradiation energy effect on GA cytotoxicity

**Fig. 4:**
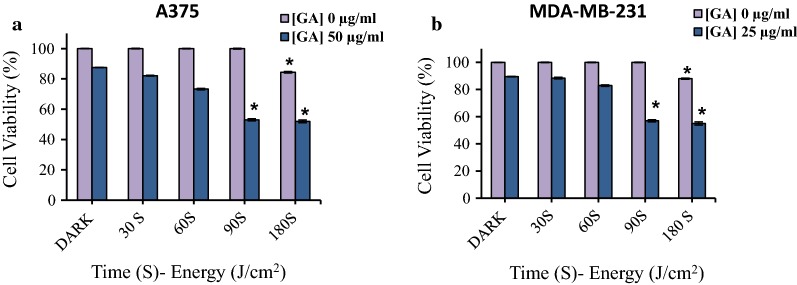
The effect of various pre-irradiation energy on GA cytotoxicity in A375 (**a**) and **b** MDA-MB-231 cancer cell lines. The data represent as the mean ± SD (n = 3). *P < 0.05 compared with dark group

The effect of different laser irradiation energies at 660 nm in concentrations of 0, 25 and 50 μg/ml of GA (IC_50_ of GA in pre-irradiation for MDA-MB-231 and A375 cell line, respectively) on human cancer cells was investigated. The results showed that 25 and 50 μg/ml of GA in the absence of low-level laser (dark condition) reduced the percentage of cell survival up to 87.5% in A375 and 98.5% in MDA-MB-231 cell lines. The cell viability was decreased to 82.2% (A375) and 88.4% (MDA-MB-231) at laser irradiation for 30 s and energy levels of 1 J/cm^2^. During radiation of 60 s with an energy level of 2 J/cm^2^, the cell viability was 73.3% (A375) and 82.9% (MDA-MB-231) and at a radiation of 90 s with radiation energy of 3 J/cm^2^, cell viability was 53% (A375) and 57% (MDA-MB-231). The cell viability decreased to 52% (A375) and 55% (MDA-MB-231) at 180 s of radiation with radiation energy of 6 J/cm^2^ (Fig. 4).

These results suggested that in higher energy and longer time of irradiation, cell survival was further reduced. However, at a radiation level of 180 s with radiation energy of 6 J/cm^2^, the cell survival rate of the control also decreased to 84.5% (A375) and 88% (MDA-MB-231). According to the results, the optimal radiation time and energy for the next studies were considered to be 90 s with radiation energy of 3 J/cm^2^.

### Cell death mechanism evaluation: microscopy and flow cytometry analysis

**Fig. 5:**
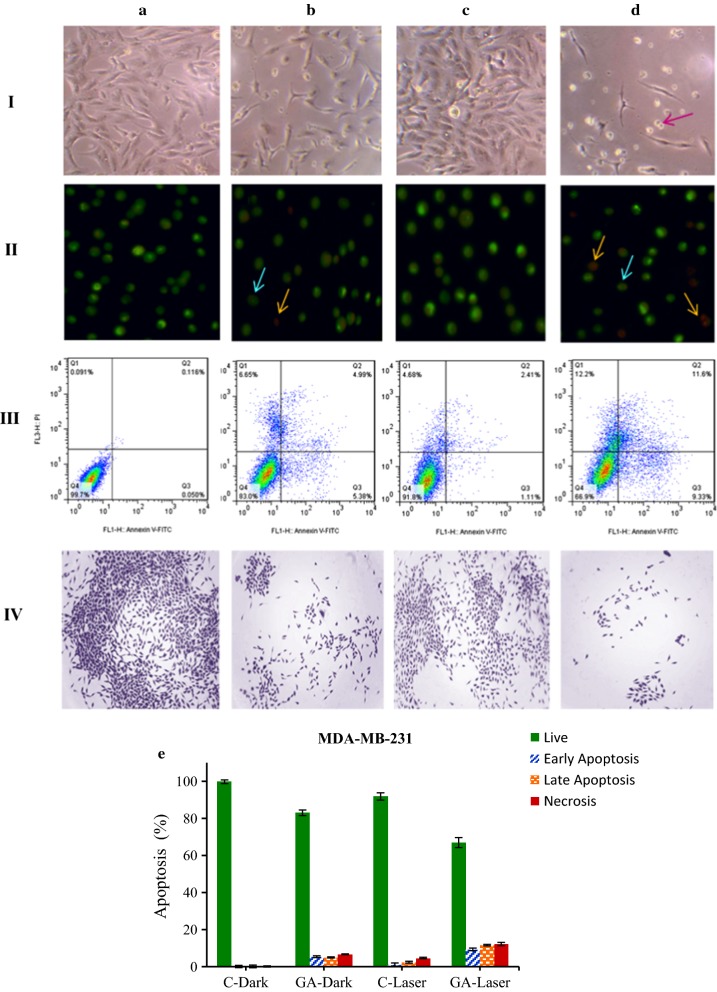
Microscopy and Flow cytometry analysis of MDA-MB-231 cells after pre-irradiation and treatment with GA for 24 h. panel I: invert microscopy images (×40), panel II: AO/EB double staining, panel III: the apoptotic rates (annexin V-FITC/PI dual staining) and panel IV: colony-forming ability. **a** 0 µg/ml (control), and **b** 25 µg/ml of GA at dark condition, **c** 0 µg/ml and **d** 25 µg/ml of GA under low-level laser irradiation. **e** Histogram showing percentages of apoptotic and necrotic cells in the dark and irradiation groups treated with irradiation and GA. Percent of apoptotic and necrotic cells obtained from (panel III)

To observe the effect of low-level laser irradiation on the morphology of both cancer cells in the presence of GA and irradiation, the cells were irradiated with 660 nm laser irradiation at 3 J/cm^2^ energy and then treated different concentrations of GA (0 and IC_50_) for 24 h. The cells were studied by invert light microscopy (40×). As shown in Fig. 5 panel I, the A and B sections represent MDA-MB-231 cells in 0 and 25 µg/ml of GA at the dark condition and, C and D sections show breast cancer cells in 0 and 25 µg/ml of GA under irradiation, respectively. As can be seen by adding GA concentration at 25 μg/ml and in the presence of irradiation, the number of cells remarkably decreased as well as the morphology of the cells changed from spindle to rounded shape.

Panel II of Fig. 5; show the morphological changes of MDA-MB-231 cells using AO/EB dual staining by fluorescence microscopy. As it can be seen in the control group (0 μg/ml of GA), the cells represent the shape of live cells with green color. By adding GA at 25 μg/ml, the nuclei of cells change to orange-red cells show early/late apoptosis. Under irradiation and in the presence of GA, the breast cancer cells show the characteristic of apoptotic cells with chromatin condensation and nuclear fragmentation. It suggests that in the presence of GA under irradiation, the cells intend to death. For understanding the death mechanism in each condition, the flow cytometry assay with annexin/PI was performed. As can be seen in panel III of Fig. 5, by adding the GA and also in the presence of irradiation, the number of apoptotic cells in early or late stages more increased in comparison to alone GA group or control. Panel IV represents the colony-forming ability of MDA-MB-231 breast cancer cells in the presence of irradiation and then GA, as it has seen the colonies were further decreased compared to alone GA group or control.

**Fig. 6:**
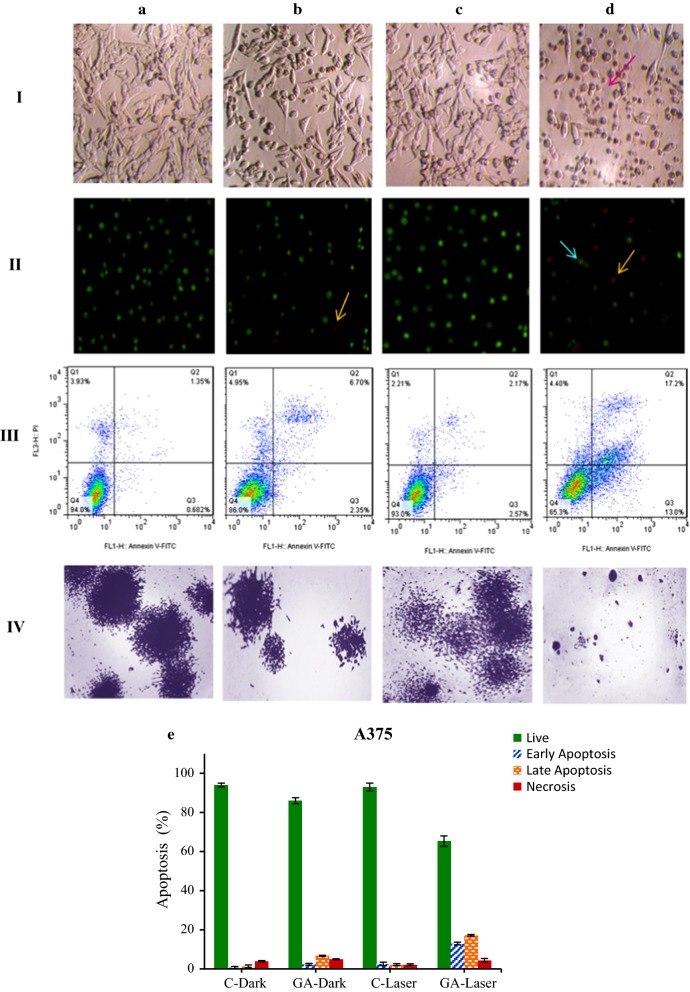
Microscopy and Flow cytometry analysis of A375 melanoma cancer cells after pre-irradiation and treatment with GA for 24 h. Panel I: invert microscopy images (×40), panel II: AO/EB double staining, panel III: the apoptotic rates (annexin V-FITC/PI dual staining) and panel IV: colony-forming ability. **a** 0 µg/ml (control), and **b** 25 µg/ml of GA at dark condition, **c** 0 µg/ml and **d** 25 µg/ml of GA under low-laser irradiation. **e** Histogram showing percentages of apoptotic and necrotic cells in the dark and irradiation groups treated with irradiation and GA. Percent of apoptotic and necrotic cells obtained from (panel III)

In the case of A375 melanoma cancer cells, as can be seen in the panel I of Fig. 6, the cells treated with GA under irradiation turn to round shape and showed the dead cell morphology. In fluorescence microscopy images, the cells represent the features of apoptotic cells with orange to red color and live cells (green) is more decreased upon irradiation at 25 μg/ml of GA (panel II) as compared with alone GA group or control. The flow cytometry analysis confirmed the microscopy result that under red irradiation and treatment with 25 μg/ml of GA, the apoptotic cells are increased in compared to alone GA group or control (panel III). As presented in panel IV, the colony-forming ability of cells treated with irradiation and GA was more reduced in comparison to alone GA group or control.

### ROS production in cancer cells after irradiation and then treatment with GA

**Fig. 7:**
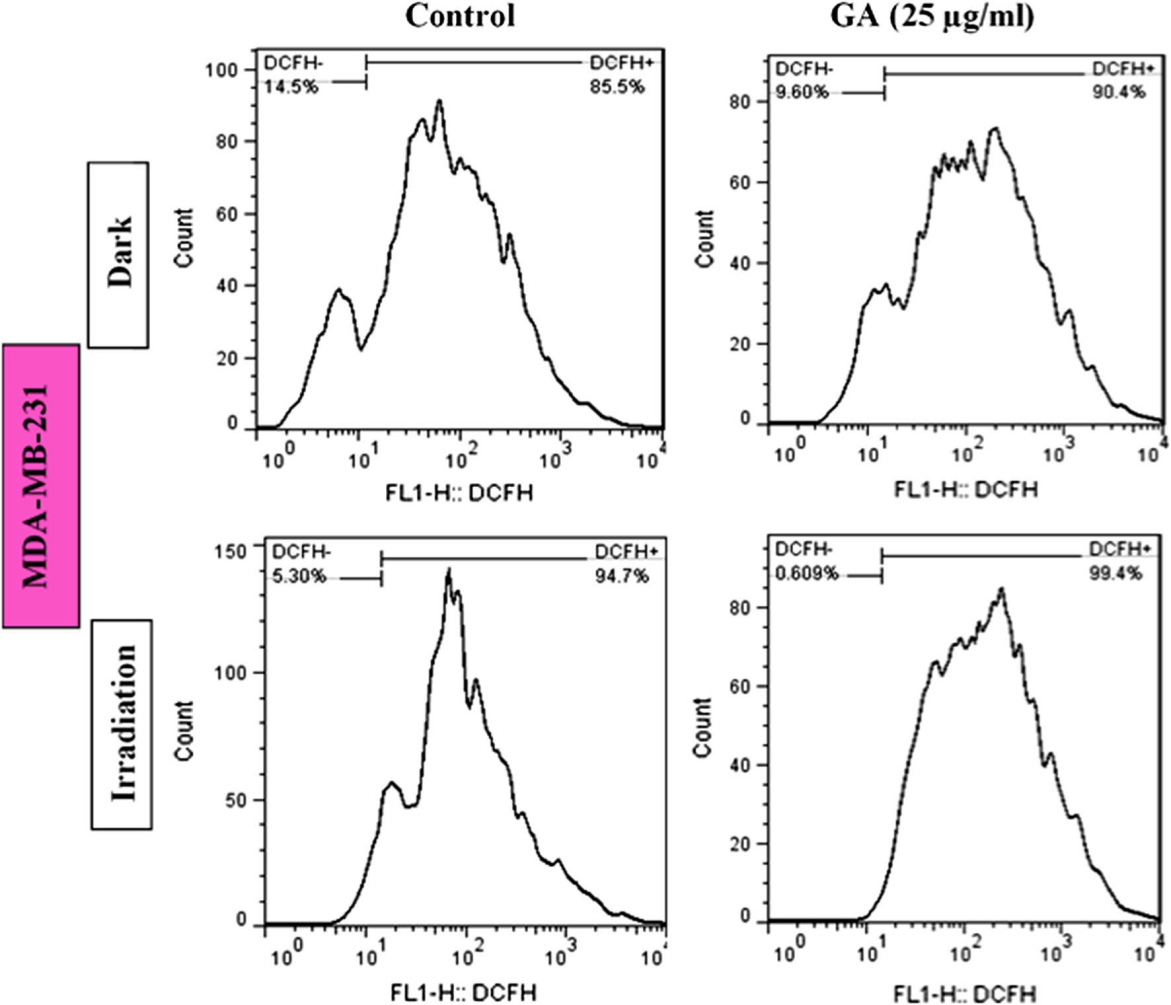
Effects of red light irradiation for 90 s and GA (25 µg/ml) on intracellular ROS generation in MDA-MB-231 cells. The cells were stained with DCFH-DA (2 mM), analyzed by flow cytometry

**Fig. 8:**
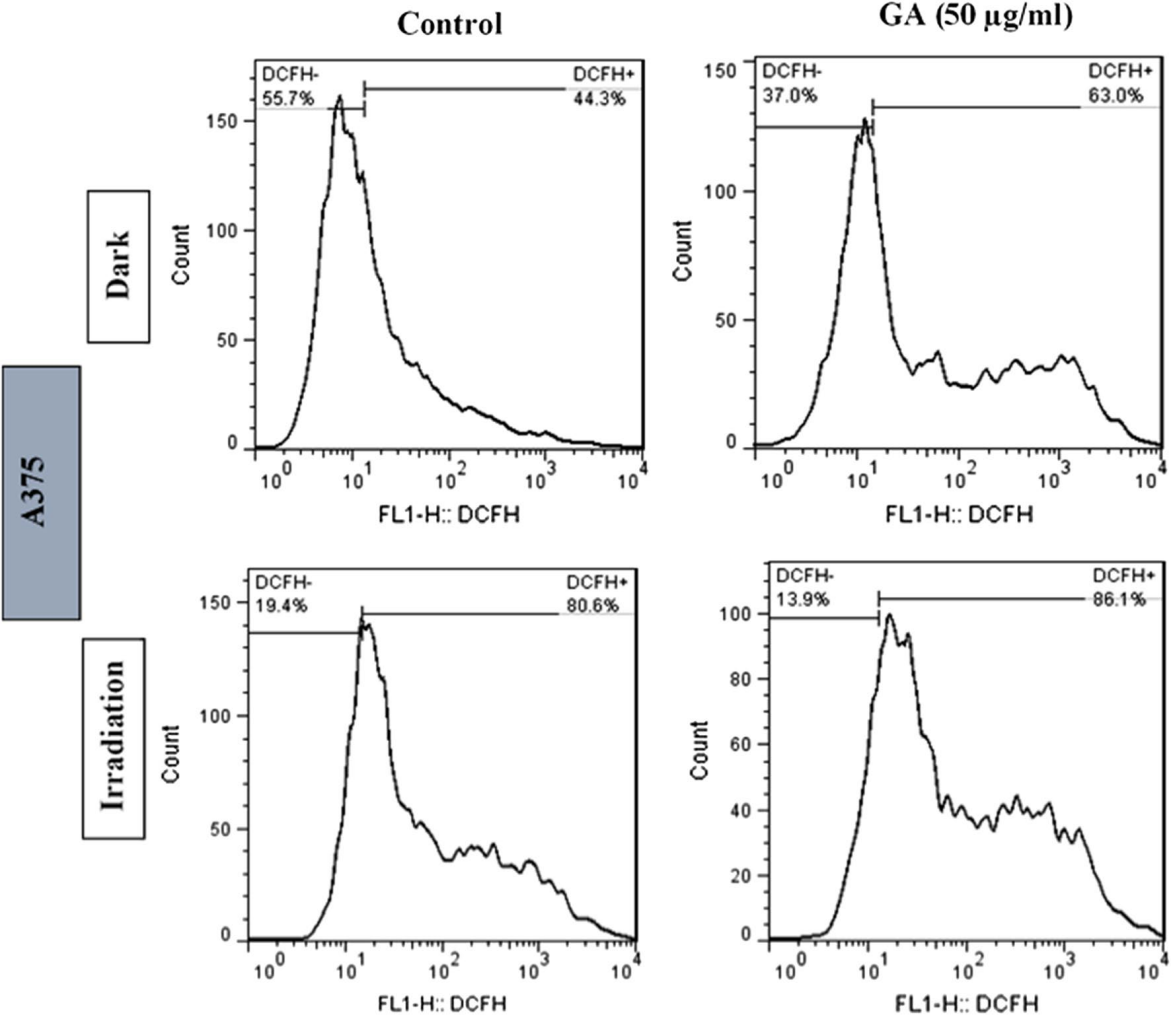
Effects of red light irradiation for 90 s and GA (25 µg/ml) on intracellular ROS generation in A375 cells. The cells were stained with DCFH-DA (2 mM), analyzed by flow cytometry

As illustrated in Figs. 7 and 8, both breast and melanoma cancer cells showed the ROS production after treatment with GA in compare to the control group (0 µg/ml). In the presence of irradiation and then adding the GA, the cancer cells represent a higher amount of ROS in compare to alone GA samples or control (irradiation-0 µg/ml) group. It suggests that ROS production could act as one of the main factors in the death mechanism of cancer cells by irradiation and treatment with GA.

### Evaluation of glutathione peroxidase activity

**Fig. 9:**
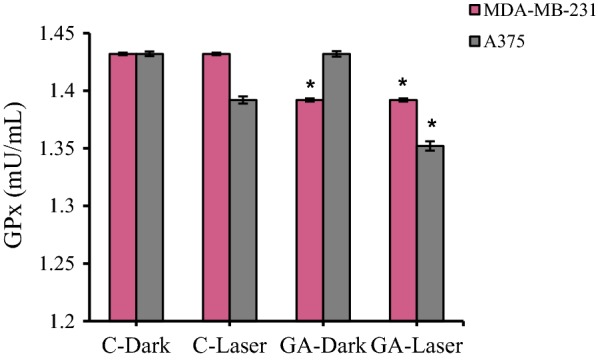
The glutathione peroxidase (GPX4) activity in MDA-MB-231 and A375 cancer cells upon low level laser irradiation and GA treatment. Low-level laser irradiation was used with 3 J/cm^2^ energy for 90 s. The results are expressed as the mean ± SD (n = 3), *P < 0.05 (compared with control groups. C-Dark: control (untreated cells in dark), C-Laser (control (untreated cells) with irradiation), GA-Dark (GA treated cells in dark) and GA-Laser (irradiation + GA treated cells)

GPX4 is one of the most important antioxidant enzymes and an essential regulator of ferroptotic cancer cell death. Recent studies suggested that the reduced level of GPX4 activity can promote ferroptosis and inflammation. Our study showed that GPX4 activity is decreased in MDA-MB-231 and A375 cancer cells as treated with low-level laser irradiation and GA compare to control groups (Fig. 9). The GPX4 activity reduced in A375 cells upon irradiation in compare dark group. After treatment with GA there is no significant reduction in GPX4 activity in compare to control dark group (C-Dark). The GPX4 activity reduced in A375 cells after treatment with irradiation plus GA in compare to control group (GA-dark and C-laser) (Fig. 9). The MDA-MB-231 cells represented the reduced GPX4 activity after treatment with GA in dark condition in compare to control dark group. GA treated MDA-MB-231 cells in presence of irradiation have shown the reduction in GPX4 activity compared to control laser group (Fig. 9).

### Lipid peroxidation measurement

**Fig. 10:**
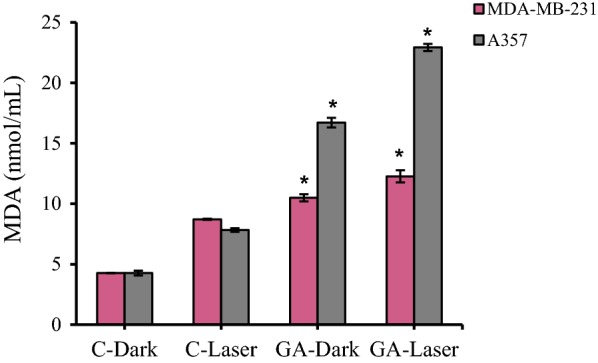
The MDA production in MDA-MB-231 and A375 cancer cells upon low level laser irradiation and GA treatment. Low-level laser irradiation was used with 3 J/cm^2^ energy for 90 s. The results are expressed as the mean ± SD (n = 3), *P < 0.05 (compared with control groups. C-Dark: control (untreated cells in dark), C-Laser (control (untreated cells) with irradiation), GA-Dark (GA treated cells in dark) and GA-Laser (irradiation + GA treated cells)

Levels of malondialdehyde (MDA), an end-product of lipid peroxides, can replace lipid peroxides as a biomarker in ferroptosis [37]. MDA production was evaluated in MDA-MB-231 and A375 cancer cells following irradiation and GA treatment. As can be seen in Fig. 10, The MDA production increased in A375 cells upon irradiation in compare dark group. After treatment with GA there is significant increasing in MDA production in compare to control dark group (C-Dark). The MDA production increased in A375 cells after treatment with irradiation plus GA in compare to control group (GA-dark and C-laser) (Fig. 10). The MDA-MB-231 cells represented the enhanced MDA production after treatment with GA in dark condition in compare to control dark group. GA treated MDA-MB-231 cells in presence of irradiation have shown the increasing MDA production compared to control laser group (Fig. 10).

## Discussion

There are many different ways to treat cancer such as surgery, chemotherapy, radiation therapy and hormone therapy. One of the biggest limitations of anticancer drugs is the resistance of cancer cells to the drug, which can be due to the intrinsic resistance of the tumor to the drug or acquired during chemotherapy also making the treatment more difficult as the resistant cells grow [38, 39]. Nowadays, herbal-based drug has received more attention than chemical drugs due to their low side effects [40]. GA is one of the known polyphenols in plants and an important antioxidant compound against cancer. Studies have shown that GA is effective in the treatment of pancreatic, colon, breast and melanoma cancers [16, 41–43]. Here, our results indicate that GA has cytotoxicity against A375 and MDA-MB-231 cell line and the IC_50_ for melanoma A375 cell line is higher than and MDA-MB-231 cell line and other cell lines which previously reported. This result may be because of an aggressive form of melanoma malignant cells. Also, our result in agreement with previous reports revealed that GA has dose-dependent cytotoxicity on cancer cells [44].

According to various reports about the effect of laser on normal and cancerous cells, it has been found that the laser effect is not identical and constant and depends on the associated treatments. The results of this study also showed that pre-treatment of human breast MDA-MB-231 and human melanoma A375 cancer cells with low-level laser and then treatment of these cells with GA suppresses survival and growth of both cancer cells more than treatment with alone GA. This result is in agreement with our previous study that para coumaric acid has more cytotoxicity on melanoma cancer cells in the presence of pre-irradiation [21].

This study showed that low-level laser alone is not capable of killing breast and melanoma cancer cells, but the use of low-level laser can somehow improve the cellular penetration of breast and melanoma cells into GA. As a result, its anticancer effect increases.

Morphological observations of cells irradiated and treated with GA confirmed the findings of the cell viability study. The cell death rate in cells exposed to low-level laser and then GA was higher than in cells treated with GA alone (the dark condition). Our study also showed that simultaneous treatment with low-level laser and then GA increased the amount of ROS produced in both breast and melanoma cancer cells compared to the alone GA-treated cell, which could induce more cell death compare to alone GA.

In addition, our study on low-level laser irradiation and GA effect on cell death showed that low-level laser treatment and then GA treatment increased the rate of cell death. Also, there is an increase in apoptotic death in human melanoma A375 more than MDA-MB-231 breast cancer cells.

Ferroptosis is a genetically programmed iron-dependent form of regulated cell death driven by enhanced lipid peroxidation and insufficient capacity of thiol-dependent mechanisms (glutathione peroxidase 4, GPX4) to eliminate hydroperoxy-lipids.

GPX4 activity was analyzed in cancer cells treated with low-level laser irradiation and GA. The results show that the loss of GPX4 activity by low level laser irradiation and GA treatment may related to induction of ferroptosis in in MDA-MB-231 breast and A375 melanoma cancer cells.

Lipid peroxidation has been identified to be directly involved in mediating necrosis and ferroptosis [17]. We next investigated whether low-level laser irradiation and GA treatment affected the production of malondialdehyde (MDA; an end product of lipid peroxidation). As it can be seen from results (Fig. 10), the low level laser irradiation and GA treatment was involved in induction of ferroptosis in both MDA-MB-231 breast and A375 melanoma cancer cells.

A recent study by Tang and Cheung [45] demonstrated that GA could induce coexistence of multiple types of cell death pathways, including apoptosis characterized by mitochondrial cytochrome c release and caspase-3 activation, ferroptosis characterized by lipid peroxidation, and necroptosis characterized by the loss of plasma membrane integrity [45]. Our study is in consistent with their study that GA could have anticancer effect via different cell death mechanism and also we found that the cell death mechanisms of GA could be potentiate by using low-level laser irradiation as pre-treatment.

## Conclusion

Taken together, our study suggests that low-level irradiation alone is not able to kill human breast and melanoma cancer cells but using pre red laser irradiation could improve cellular penetration of GA and consequently improve its anticancer effects possibly through inducing apoptosis and ferroptosis pathway mainly via ROS production, decreasing GPX4 activity and increasing Lipid peroxidation.

Although our research implies new strategies to enhance the efficacy of gallic acid as anti-cancer compound in cancer treatment at the cellular level, the precise mechanism remains unknown and should be elucidated.

## Data Availability

The datasets generated and analyzed during the current study are available from the corresponding authors on reasonable request by permission of institute and department chairman’s.
